# RecQL4-Aurora B kinase axis is essential for cellular proliferation, cell cycle progression, and mitotic integrity

**DOI:** 10.1038/s41389-018-0080-4

**Published:** 2018-09-12

**Authors:** Hongbo Fang, Kaifeng Niu, Dongliang Mo, Yuqi Zhu, Qunsong Tan, Di Wei, Yueyang Li, Zixiang Chen, Shuchen Yang, Adayabalam S. Balajee, Yongliang Zhao

**Affiliations:** 1 Key Laboratory of Genomic and Precision Medicine, Beijing Institute of Genomics, Chinese Academy of Sciences, 100101 Beijing, China; 2 University of Chinese Academy of Sciences, 100049 Beijing, China; 3 Cytogenetics Biodosimetry Laboratory, REACTS, Oak Ridge Institute for Science and Education, Oak Ridge Associated Universities, 1299 Bethel Valley Road, Oak Ridge, TN 37830 USA

## Abstract

Human RecQL4 helicase plays critical roles in the maintenance of genomic stability. Mutations in RecQL4 helicase results in three clinically related autosomal recessive disorders: Rothmund–Thomson syndrome (RTS), RAPADILINO, and Baller–Gerold syndrome. In addition to several premature aging features, RTS patients are characterized by aneuploidy involving either loss or gain of a single chromosome. Chromosome mosaicism and isochromosomes involving chromosomes 2, 7, and 8 have been reported in RecQL4-deficient RTS patients, but the precise role of RecQL4 in chromosome segregation/stability remains to be elucidated. Here, we demonstrate that RecQL4 physically and functionally interacts with Aurora B kinase (AURKB) and stabilizes its expression by inhibiting its ubiquitination process. Our study indicates that the N-terminus of RecQL4 interacts with the catalytic domain of AURKB. Strikingly, RecQL4 suppression reduces the expression of AURKB leading to mitotic irregularities and apoptotic cell death. RecQL4 suppression increases the proportion of cells at the G2/M phase followed by an extensive cell death, presumably owing to the accumulation of mitotic irregularities. Both these defects (accumulation of cells at G2/M phase and an improper mitotic exit to sub-G1) are complemented by the ectopic expression of AURKB. Finally, evidence is provided for the requirement of both human telomerase reverse transcriptase and RecQL4 for stable immortalization and longevity of RTS fibroblasts. Collectively, our study suggests that the RecQL4–AURKB axis is essential for cellular proliferation, cell cycle progression, and mitotic stability in human cells.

## Introduction

Human RecQL4 helicase plays multifaceted roles in the maintenance of genomic stability and mutations in RecQL4 leading to three autosomal recessive disorders: Rothmund–Thomson syndrome (RTS), RAPADILINO syndrome, and Baller–Gerold syndrome (BGS), and these three syndromes are somewhat clinically related^1^. While type I RTS patients are free of RecQL4 mutations, type II patients are often characterized by RecQL4 mutations with an increased risk for osteosarcoma development^2,3^. Cells of RTS patients show retarded proliferation in vitro emphasizing a critical role for RecQL4 in DNA replication. Recent studies have demonstrated that RecQL4 protects the integrity of nuclear and mitochondrial genomes through its interaction with some of the proteins involved in genome surveillance and DNA repair^4,5^. One of the characteristic cellular features of RecQL4-deficient RTS patients is aneuploidy with a loss or gain of a chromosome resulting in an abnormal diploid number of 45 or 47 chromosomes instead of 46 chromosomes^2,6,7^. Aneuploidy is considered to be due to mal-segregation of chromosomes in either of the gametes during meiosis. Mosaicism involving chromosomes 2, 7, and 8 have been reported in the cells of RTS patients and chromosome mosaicism is due to chromosome segregation error occurring after zygote formation and initiation of cell division^8^. Collectively, these defects in RTS patients indicate a pivotal role for RecQL4 in chromosome segregation process. Strikingly, testis is one of the organs in humans with the highest level of RecQL4 expression^9^ and it is highly probable that RecQL4 deficiency may lead to aberrant meiosis. Mitosis is a crucial phase in cell cycle where the replicated chromosomes segregate properly between two daughter nuclei in somatic cells. Any disruption in chromosome segregation is likely to result in mitotic catastrophe causing cell death. Cancer cells overcome the mitotic catastrophe by achieving an increased expression for some of the pro-survival proteins including Survivin^10^.

When cells are challenged with DNA damage, a transient cell cycle arrest, based on the extent of DNA damage, is imposed at G1, S, and G2/M phases, thereby ensuring the completion of DNA repair process^11^. Among the cell cycle phases, G2/M phase is considered to be most sensitive to certain agents such as ionizing radiation and the duration of G2/M arrest after radiation exposure is dose dependent^12,13^. When cells are exposed to an extensive DNA damage, mitotic catastrophe can be triggered by several factors, such as DNA damage persistence, disruption of mitotic spindles, prolonged growth arrest, and inhibition of cyclin-dependent kinases^14^. An efficient mitotic spindle assembly, which is essential for error free chromosome segregation, involves the chromosome passenger complex (CPC), composed of inner centromere protein (INCENP), Survivin (also known as BIRC5), Borealin, and Aurora B kinase (AURKB). This complex regulates key mitotic events, including the correction of chromosome-microtubule attachment and activation of the spindle assembly checkpoint^15–17^. RecQL4 physically interacts with Survivin^18^. Importantly, Survivin and AURKB proteins participate in one of the anti-apoptotic pathways^14^.

Cells of RTS patients show not only chromosome aneuploidy but also premature replicative senescence^4^. It is likely that the replicative senescence is triggered by a telomere loss driven by an increased accumulation of DNA damage at the telomeres. In support, RecQL4 has been demonstrated to protect the telomere stability by unwinding of damage containing telomeric D-loops through interaction with telomere regulatory proteins: TRF1, TRF2, and POT1^19^. Therefore, mutational inactivation of RecQL4 can cause telomere instability leading to replicative senescence. Detection of chromosome mosaicism and isochromosomes in RTS patients suggests a potential role for RecQL4 in chromosome segregation. Consistent with this, aneuploidy and premature chromatid separation were also reported in RecQL4 knockout mice. Increased expression of RecQL4 reported in G2 and M phases^20^ strengthens the assumption that RecQL4 is essential for chromosome stability during different mitotic phases. However, the mechanistic basis for the cause of chromosome aneuploidy and mosaicism in RecQL- deficient RTS cells is not yet fully explored. AURKB, a central component of CPC complex, has been shown to be a key player in chromosome segregation^15^. Suppression of AURKB led to failure of chromosome bi-orientation, leading to massive polyploidy and cell death^21,22^. In contrast, AURKB upregulation has been associated with poor prognosis in glioblastoma, ovarian carcinoma, and hepatocellular carcinoma^23^. Since RecQL4 deficiency causes chromosome segregation defects in RTS patients, we wished to determine the underlying mechanism for RecQL4 in maintaining chromosomal stability. Our results indicate that RecQL4 physically and functionally interacts with AURKB and stabilizes AURKB by inhibiting its ubiquitination. Suppression of RecQL4 resulted in a substantial reduction in AURKB expression owing to its increased degradation by ubiquitination. As expected, reduced AURKB expression caused by RecQL4 suppression led to a severe mitotic catastrophe. Further, RecQL4-suppressed cells displayed an extensive death owing to mitotic catastrophe, which was rescued by the ectopic expression of AURKB. Collectively, our findings suggest that both RecQL4 and AURKB are on the same axis for protecting the chromosomal stability through concerted regulation of cellular proliferation and cell cycle progression.

## Results

### RecQL4 physically interacts with AURKB kinase

**Fig. 1:**
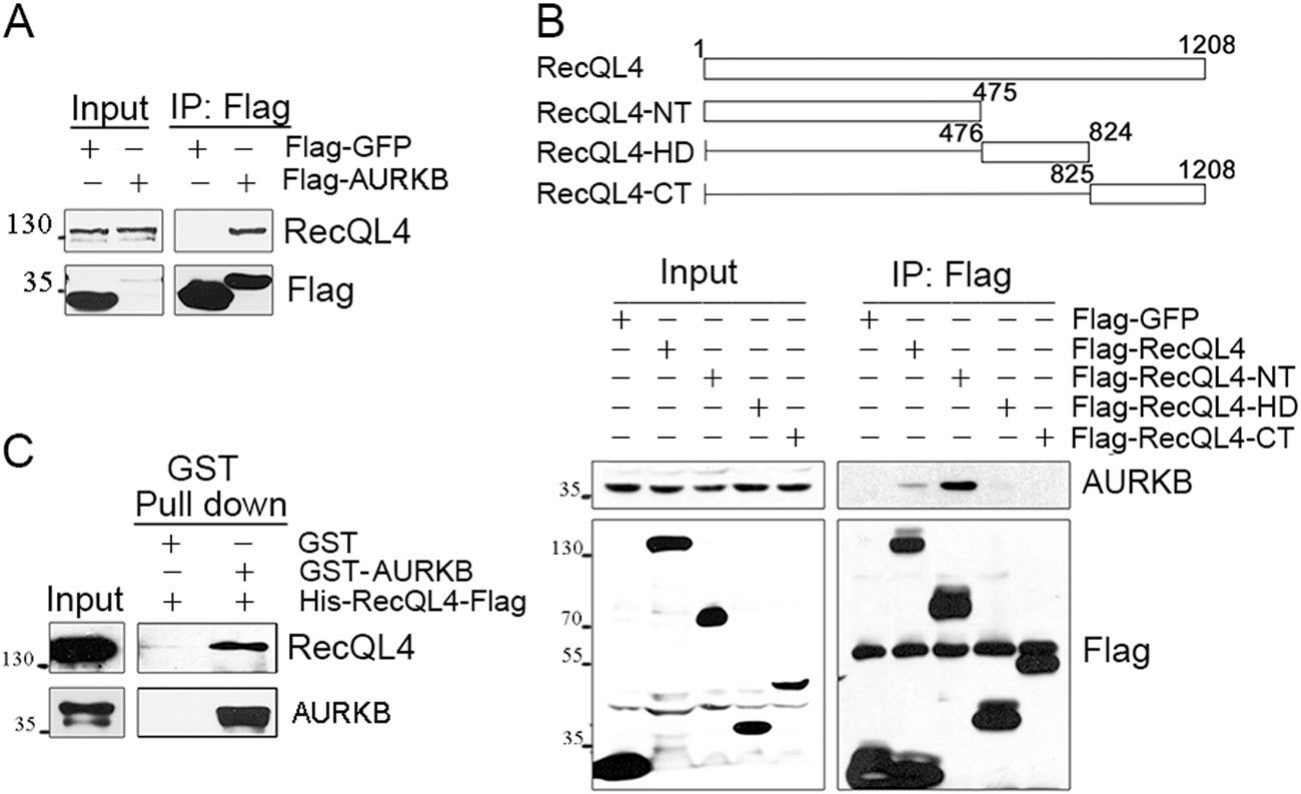
RecQL4 directly interacts with AURKB. **a** Endogenous RecQL4 was detected in anti-Flag pull-down against Flag-AURKB. Flag-GFP immnoprecipitate was used as control. **b** Endogenous AURKB was detected in anti-Flag-RecQL4 immnoprecipitate determined by Western blotting (lane 2 of Flag IP), and only N-terminus of RecQL4 (Flag-RecQL4-NT, lane 3 of Flag IP) showed an interaction with AURKB. Flag-GFP immunoprecipitate was used for negative control. **c** Direct interaction between RecQL4 and AURKB was shown by in vitro GST pull-down assay

The inherent chromosomal instability manifested as mosaicism and isochromosomes in RecQL4-deficient RTS patients prompted us to identify some of the downstream targets of RecQL4. In our earlier study, we identified Survivin as one of the interaction partners of RecQL4^18^ and as one of the key proteins in the CPC, which also includes INCENP, Borealin, and AURKB kinase, and all of these proteins play essential roles in chromosome stability^15^. Strikingly, immunoprecipitation coupled with mass spectrophotometry identified AURKB as one of the prominent interacting partners for RecQL4. To verify their association, immunoprecipitation was performed using the whole-cell lysates of 293T cells expressing either a Flag-tagged full-length RecQL4 or AURKB protein. We chose 293T cells for the immunoprecipitation assay to test protein–protein interaction mainly because 293T cells have a very high transfection efficiency (>80%) that can minimize the experimental variations arising from different transfection efficiencies on exogenous gene expression. When Flag-AURKB was precipitated with anti-Flag antibody, a band corresponding to the native RecQL4 protein (~133 kDa) was detected with anti-RecQL4 antibody, demonstrating the physical interaction of AURKB with RecQL4 (Fig. 1a). Likewise, endogenous AURKB protein was detected in the sample immunoprecipitated with anti-Flag antibody in 293T cells expressing the Flag-RecQL4 (Fig. 1b, lane 2 of Flag IP). These reciprocal immunoprecipitation experiments clearly demonstrate the physical association of RecQL4 with AURKB in 293T cells.

To precisely identify the interacting domain of RecQL4 with AURKB, all the three domains (amino terminal domain, helicase domain, and carboxyl terminal domain) designated as Flag-RecQL4-NT, Flag-RecQL4-HD, and Flag-RecQL4-CT containing an in-frame addition of Flag tag at the amino-terminus of RecQL4 were generated (Fig. 1b, upper panel). The cell lysates prepared from 293T cells transfected with each of the domain-specific expression plasmid were incubated with Flag M2 beads. A stronger interacting protein band corresponding to AURKB was detected in the precipitates of Flag-RecQL4-NT domain expressing cells, suggesting that the N-terminus of RecQL4 protein has a stronger interaction with AURKB protein (Fig.1b, lower panel). Additionally, the AURKB CAT domain was demonstrated to interact with RecQL4 (Fig. S1).

The nature of interaction between RecQL4 and AURKB was further determined by in vitro GST pull-down assay using purified His-RecQL4-Flag and GST-AURKB expressed in *Escherichia coli* BL21. GST-AURKB protein was immobilized on glutathione-Sepharose beads and then incubated with purified human His-RecQL4-Flag protein. The pull-down complexes were then analyzed by Western blot analysis using the RecQL4 antibodies. RecQL4 was observed in the pull-down complex precipitated by GST-AURKB but not by GST alone, illustrating a direct interaction between RecQL4 and AURKB (Fig. 1c).

### RecQL4 and AURKB are upregulated in various immortalized cell lines

**Fig. 2:**
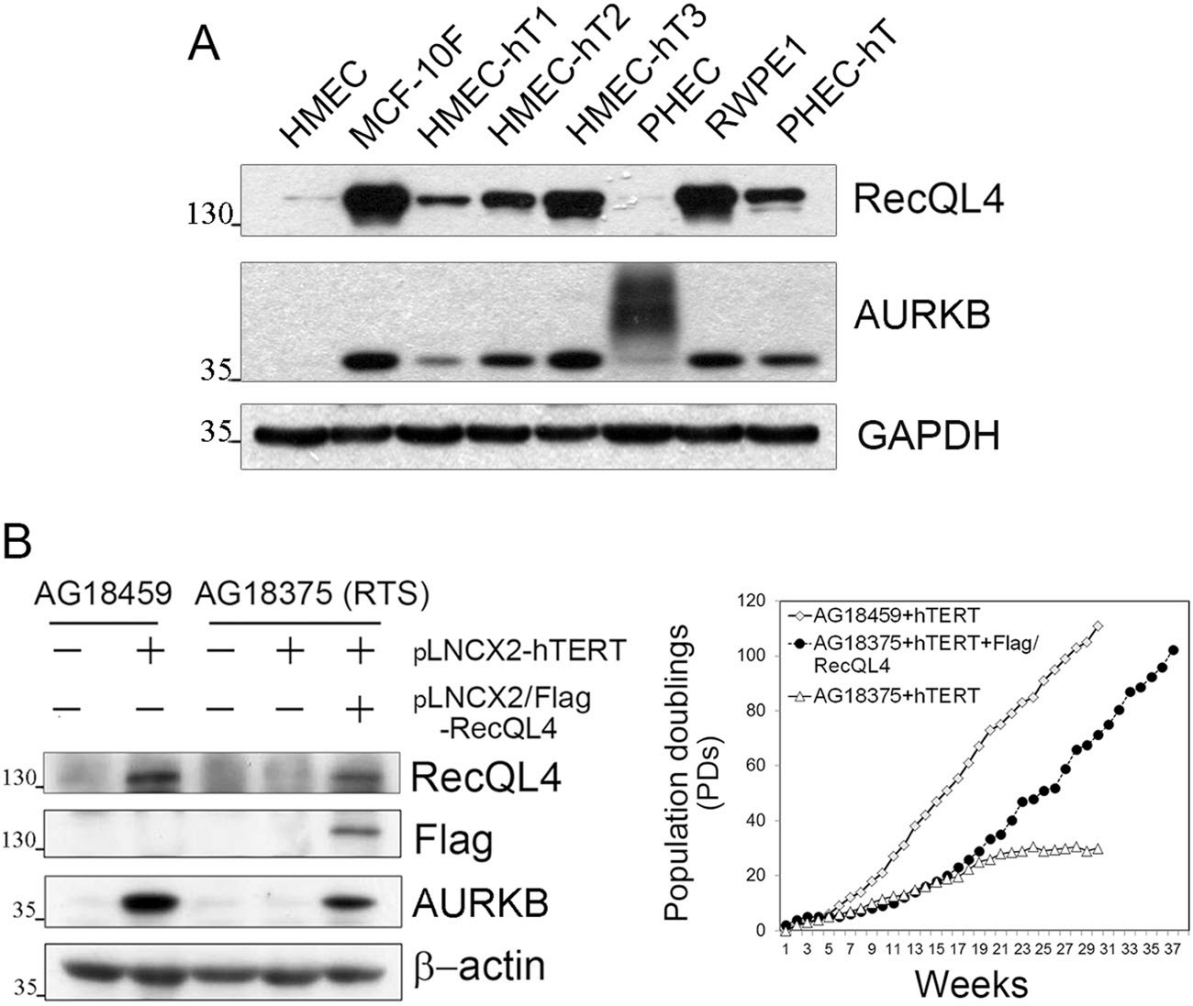
Elevated RecQL4 and AURKB expression in immortalized cell lines. **a** Expression level of RecQL4 and AURKB in spontaneous (MCF-10F), SV40-immortalized (RWPE1), and hTERT-immortalized (HMEC-hT1–3 and PHEC-hT) cells relative to their controls. HMEC human normal mammary epithelial cells, PHEC human prostatic epithelial cells. **b** RecQL4 and AURKB levels in primary and immortalized AG19459 and AG18375 cells determined by Western blotting, and population doublings (PDs) of AG18359 and AG18375 cells infected with hTERT retrovirus only, and AG18375 cells infected with both hTERT and Flag-RecQL4 retrovirus

RecQL4-deficient human fibroblasts show retarded growth potential and attain premature replicative senescence after a few population doublings (PDs) in vitro. In corroboration, fibroblasts derived from RecQL4 knockout mice also showed replicative senescence emphasizing a critical role for RecQL4 in cellular proliferation. To verify this, we investigated the expression of RecQL4 in various human cell lines immortalized by different methods: (I) spontaneous MCF-10F, (II) human telomerase reverse transcriptase (hTERT)-immortalized human mammary epithelial cells (HMEC-hT1–3) (Fig. S2) and human prostate epithelial cells (PHEC-hT)^24^, and (III) Simian virus 40 (SV40) large T-immortalized prostate epithelial cells (RWPE1). Among the three RecQ helicases examined at the mRNA level (BLM, WRN, and RecQL4), only RecQL4 expression was consistently elevated in all the immortalized cell lines (Fig. S3). The AURKB expression was also confirmed to be elevated in all those cell lines. Furthermore, AURKB level showed a clear dependency on RecQL4 expression. In particular, primary normal prostate epithelial cells (PHECs) had a low level of RecQL4 versus an enhanced AURKB ubiquitination (Fig. 2a).

### RecQL4 is required for immortalization of RTS fibroblasts

Consistent elevation of RecQL4 expression in various immortalized human cells prompted us to investigate whether or not RecQL4 is pivotal for cellular immortalization process. To verify the role of RecQL4 in immortalization process, human fibroblasts isolated from RecQL4 heterozygous mother (AG18459) and homozygous son (AG18375) were transduced with control and hTERT-expressing retrovirus vectors. The donor subject (AG18375) was a compound heterozygote having a G to A substitution (g.2626G>A) in one allele in exon 8 with a disrupted splicing (the same mutation found in AG18459) and a 1-bp deletion at g.2886 (g.2886delT) in exon 9 of the other allele leading to truncated protein. Both these mutations map to the helicase domain of RecQL4. AG18459 and AG18375 cells infected with control vector showed a finite life span of ∼4–6 PDs in culture. However, ectopic expression of hTERT resulted in an enhanced proliferative potential of both cell lines. Specifically, ectopic expression of hTERT in the fibroblasts derived from RecQL4 heterozygous carrier (A18459) extended their life span beyond ∼110 PDs (Fig. 2b). In contrast, hTERT-expressing fibroblasts derived from the son (AG18375) with homozygous mutations showed a life span of only ∼30 PDs (Fig. 2b), suggesting that these cells are somewhat refractory to immortalization in the absence of RecQL4. However, concurrent expression of both RecQL4 and hTERT in AG18375 cells led to a significantly prolonged life span of over 110 PDs (Fig. 2b). This finding unequivocally establishes the requirement of RecQL4 for a successful hTERT-mediated immortalization of RTS fibroblasts. Interestingly, elevated levels of both RecQL4 and AURKB were observed in AG18459 cells after ectopic expression of hTERT, indicating a possibility that hTERT acts upstream of both RecQL4 and AURKB.

### RecQL4 stabilizes AURKB by inhibiting its degradation

**Fig. 3:**
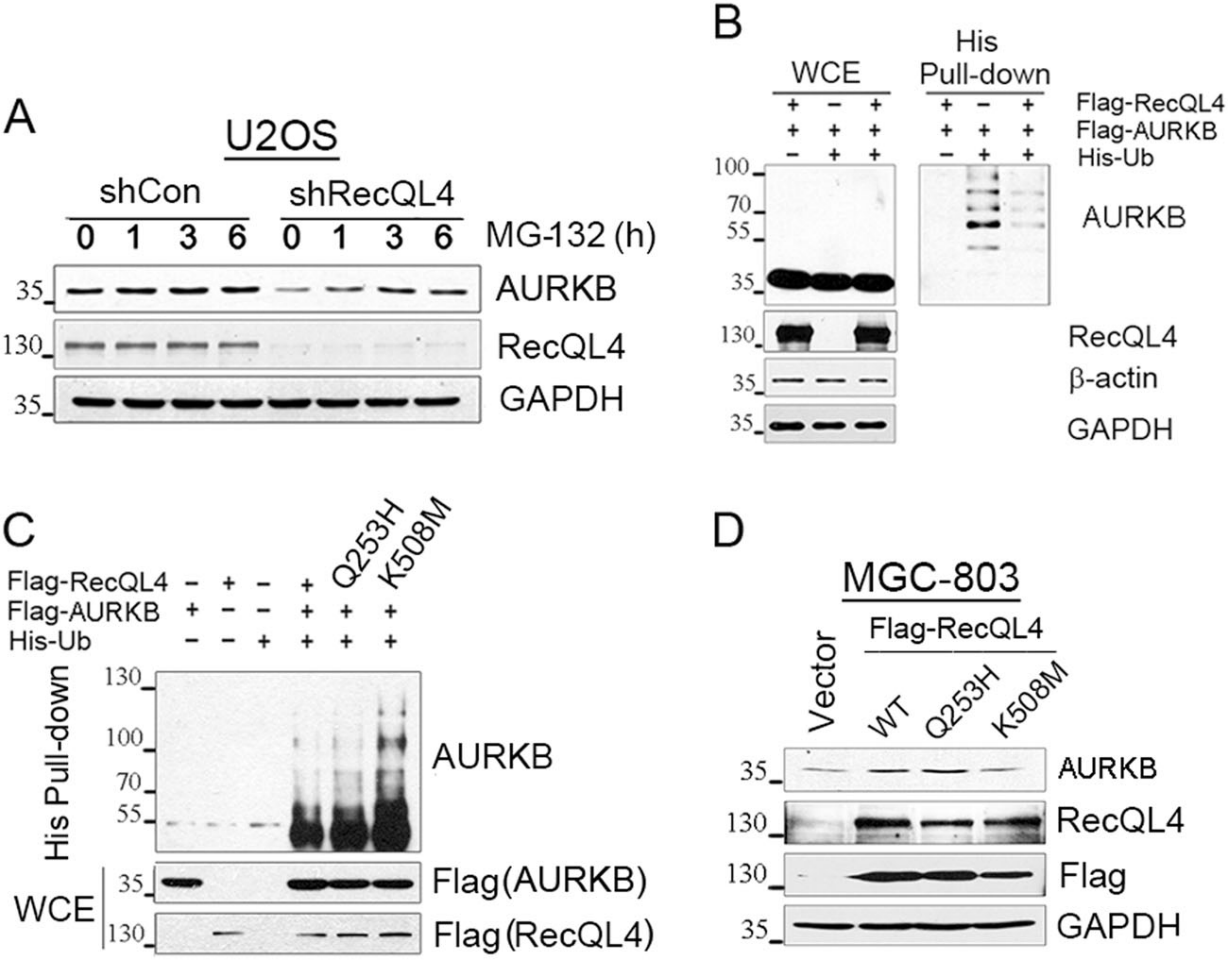
RecQL4 regulates AURKB stability. **a** Enhanced proteasome-dependent degradation of AURKB post RecQL4 knockdown. RecQL4 was first silenced by adenovirus-mediated RecQL4 shRNA, and then treated with MG-132 for 1, 3, and 6 h. Cell lysates were harvested and levels of AURKB and RecQL4 were determined by Western blotting. **b** Decreased ubiquitination level of AURKB in the presence of RecQL4. RecQL4 was first silenced in U2OS cells and then transfected with His-Ub and Flag-AURKB currently with or without Flag-RecQL4 reconstitution. Cells transfected with Flag-AURKB and Flag-RecQL4 without His-Ub were used as control. AURKB ubiquitination level was determined in His pull-down complex by Western blotting. **c** RecQL4 helicase domain-mutation affects AURKB ubiquitination. Native RecQL4 expression was first silenced by adenoviral RecQL4 shRNA and then reconstituted by His-Ub and Flag-AURKB with either WT RecQL4 or its mutants (Q253H/K503M). AURKB ubiquitination level was examined in His pull-down by Western blotting. **d** RecQL4 enhances AURKB protein level. MGC-803 cells expressing a low level of native RecQL4 were transfected with control vector and Flag-tagged wild-type (WT) or its mutants (Q253H/K508M) expressing vectors. AURKB protein level was determined by Western blotting

It has been reported that AURKB protein level was strictly controlled during each stage of mitosis primarily through ubiquitination-mediated degradation^25^. To test whether RecQL4 affects the degradation of AURKB, RecQL4 expression was suppressed in U2OS in the presence of a proteasome inhibitor MG-132. A markedly decreased level of AURKB protein was observed in RecQL4-silenced cells relative to short hairpin RNA (shRNA) control, whereas it was substantially elevated by treatment with MG-132 for more than 1 h (Fig. 3a), suggesting that RecQL4 knockdown promotes proteasome-mediated AURKB degradation. We then compared the ubiquitination level of AURKB under RecQL4-deficient and RecQL4-proficient conditions. Histidine pull-down assay was performed in RecQL4-suppressed U2OS cells transfected with Ub-His plasmid and Flag-AURKB with or without Flag-RecQL4 reconstitution. The observation showed that AURKB ubiquitination level was markedly suppressed in the presence of Flag-RecQL4 (Fig. 3b, lanes 2 and 3 of His pull-down). This finding clearly suggests that RecQL4 is required for the stabilization of AURKB protein through suppression of its ubiquitination-mediated degradation.

To explore the functional significance of different domains of RecQL4 in modulating AURKB protein stability, we first determined whether or not RecQL4 mutation affects the interaction between RecQL4 and AURKB. The 293T cells were transfected with wild-type RecQL4 or its mutants: N-terminal sld2 domain-mutation (Q253H) and helicase domain mutant K508M, and then native AURKB level was examined in Flag pull-down complex by Western blotting. The findings showed that Q253H mutation did not interfere with the interaction of RecQL4 with AURKB, which instead showed a higher level of interaction than that of WT RecQL4. In contrast, helicase domain mutation (K508M) led to a lower interaction of RecQL4 with AURKB relative to WT RecQL4 and Q253H mutant (Fig. S4), suggesting that RecQL4 helicase domain mutation interferes its interaction with AURKB, probably through inducing conformational change of RecQL4 protein.

The important role of RecQL4 helicase domain in modulating AURKB ubiquitination was further analyzed. Native RecQL4 expression was first silenced in U2OS cells by adenoviral RecQL4 shRNA and then reconstituted by His-Ub and Flag-AURKB with either WT RecQL4 or its mutants. We observed that AURKB ubiquitination level was similar in WT and Q253H mutant RecQL4 reconstituted cells, but markedly enhanced after K508M mutant RecQL4 reconstitution (Fig. 3c). These data clearly demonstrated that RecQL4 helicase domain is critical for modulating AURKB ubiquitination and stability. Consistently, in MGC-803 cells that showed a low level of RecQL4 protein^26^, only reconstitution of WT and Q253H mutant RecQL4, but not helicase K508M mutant, led to a substantially increased level of AURKB determined by Western blotting (Fig. 3d), further illustrating that the helicase domain of RecQL4 is responsible for AURKB protein stabilization. Elucidating the exact mechanism for the protection of AURKB by RecQL4 requires further experiments.

### Modulation of RecQL4 expression affects AURKB protein level

**Fig. 4:**
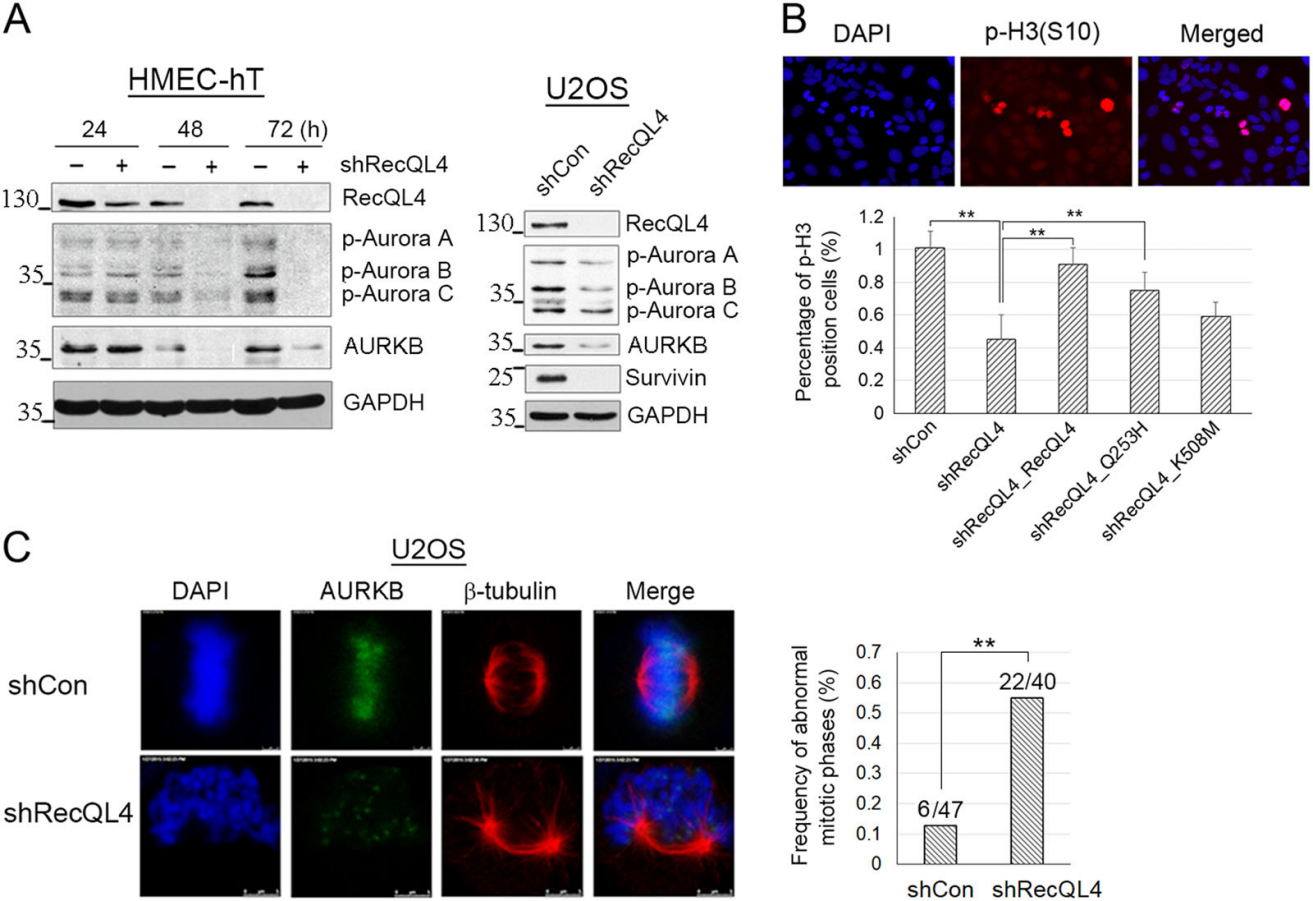
Decreased AURKB versus enhanced frequency of mitotic defect post RecQL4 knockdown. **a** Total and phosphorylation levels of AURKB were determined in HMEC-hT1 cells at 24, 48, and 72 h post RecQL4 knockdown, and in U2OS cells at 48 h post RecQL4 knockdown by Western blotting. **b**The significance of RecQL4 functional domains in mitosis. Percentage of phospho-H3(S10)-positive cells in RecQL4-silenced U2OS cells without or with reconstitution of WT or mutant RecQL4 quantified by flow cytometry. Control shRNA adenovirus-infected U2OS cells were used as control (shControl). Phosphorylated H3 was used as the marker of cells in mitotic phase. Data obtained from three independent experiments are presented as the mean ± SD. One-way ANOVA statistical analysis, **p value <0.01. **c** Aberrant mitotic phases in U2OS cells with RecQL4 depletion. Cells with lagging chromosomes and multi-spindle figures were scored as aberrant mitotic cells. Frequency of aberrant mitotic cells out of the total number of cells in mitosis was compared between shControl and shRecQL4 U2OS cells. Cells were stained for tubulin (red), AURKB (green), and DNA (blue, DAPI), and images were captured by confocal microscopy. Scale bar, 5 μm. Abnormal mitotic phase of U2OS cells post RecQL4 knockdown. *χ*^2^ test, **p < 0.01

RecQL4-mediated regulation of AURKB expression was next investigated in RecQL4-silenced hTERT-immortalized (HMEC-hT1) and malignantly transformed osteosarcoma cells (U2OS). In both cell lines, RecQL4 suppression by shRNA resulted in a consistent reduction in the levels of both non-phosphorylated and phosphorylated AURKB (Fig. 4a). Consistent with our earlier observations^18^, Survivin, a well-known substrate of AURKB, was also markedly decreased (Fig. 4a).

Activation of AURKB dependence of RecQL4 status was also observed in RTS fibroblasts (AG18375) expressing only hTERT or with RecQL4, and RecQL4 heterozygous cells (AG18459) expressing hTERT (Fig. 2b). An increased level of AURKB was observed in hTERT-immortalized AG18459 cells that contain one normal allele of RecQL4. However, RecQL4-deficicent AG18375 RTS cells expressing hTERT alone failed to show any change in AURKB expression despite a reasonable increase in life span. In contrast, RecQL4-deficient cells (AG18375) with ectopic expression of both hTERT and RecQL4 showed an elevated AURKB expression, and these cells eventually acquired an infinite replication potential in vitro judged by increased number of passages. These findings provide further evidence that AURKB functions downstream of RecQL4, and an intact RecQL4–AURKB axis is critical for cells to acquire an infinite proliferative potential. Also, our study demonstrates that RecQL4 is essential for hTERT-medicated cellular immortalization process.

### RecQL4 knockdown increases the frequency of aberrant mitotic cells

AURKB is a mitotic checkpoint kinase ensuring correct chromosome segregation and normal progression through mitosis and therefore is essential for cytokinesis^27^. Depletion of AURKB results in polyploidy probably owing to an impaired assembly of mitotic apparatus. Our finding of RecQL4-mediated regulation of AURKB expression led us to examine the impact of RecQL4 suppression on mitotic stability. We first determined the level of RecQL4 protein in different phases of cell cycle. U2OS cells were arrested at the G1 phase by double-thymidine block (DTB) and then released to enter the cell division. RecQL4 was found to be at its lowest level at the G1 phase, followed by an increase in both S and G2/M phases (Fig. S5). A substantially enhanced interaction between RecQL4 and AURKB was also observed in mitotic phase cells compared to G1 interphase cells (Fig. S6).

We further performed functional analyses about RecQL4 and its mutants by quantifying the percentage of phospho-H3 (S10)-positive cells using a rescue assay. Phosphorylated H3 (p-H3) was used as the marker of cells in mitotic phase. U2OS cells were infected with control shRNA and shRecQL4 for 24 h, and then reconstituted with WT RecQL4 or its mutants (Q253H/K508M) for 48 h. All the cells were immunostained with p-H3(S10) antibody and the percentage of p-H3-positive cells was analyzed by flow cytometry. In the Fig. 4b, the upper panel shows the representative immunostaining picture of p-H3 (S10)-positive U2OS cells counterstained with 4′,6-diamidino-2-phenylindole (DAPI) in the exponential growth phase. The lower panel shows the quantitative data obtained by flow cytometry in RecQL4-silenced U2OS cells without or with reconstitution of WT RecQL4 or its mutants (Q253H/K508M). RecQL4 knockdown led to a significantly decreased percentage of p-H3(S10)-positive cells relative to shControl, whereas reconstitution of WT or Q253H mutant RecQL4, but not K508M mutant, significantly enhanced the frequency of p-H3-positive cells. These findings illustrate an essential function of RecQL4 helicase domain in mitosis.

The significance of RecQL4 in mitosis was further validated by analyzing the abnormal mitotic phases post RecQL4 depletion in U2OS cells. As shown in Fig. 4c, AURKB staining intensity was greatly reduced in RecQL4-suppressed cells. As expected, reduced AURKB expression found in RecQL4-suppressed cells correlated well with a significantly increased frequency of aberrant mitotic cells (22/40; 55%) relative to shControl adenovirus-treated cells (6/47, 12.8%) suggestive of a positive correlation between RecQL4 and AURKB in mitotic stability.

### RecQL4 knockdown induces G2/M arrest and mitotic cell death that could be rescued by overexpressing AURKB

**Fig. 5:**
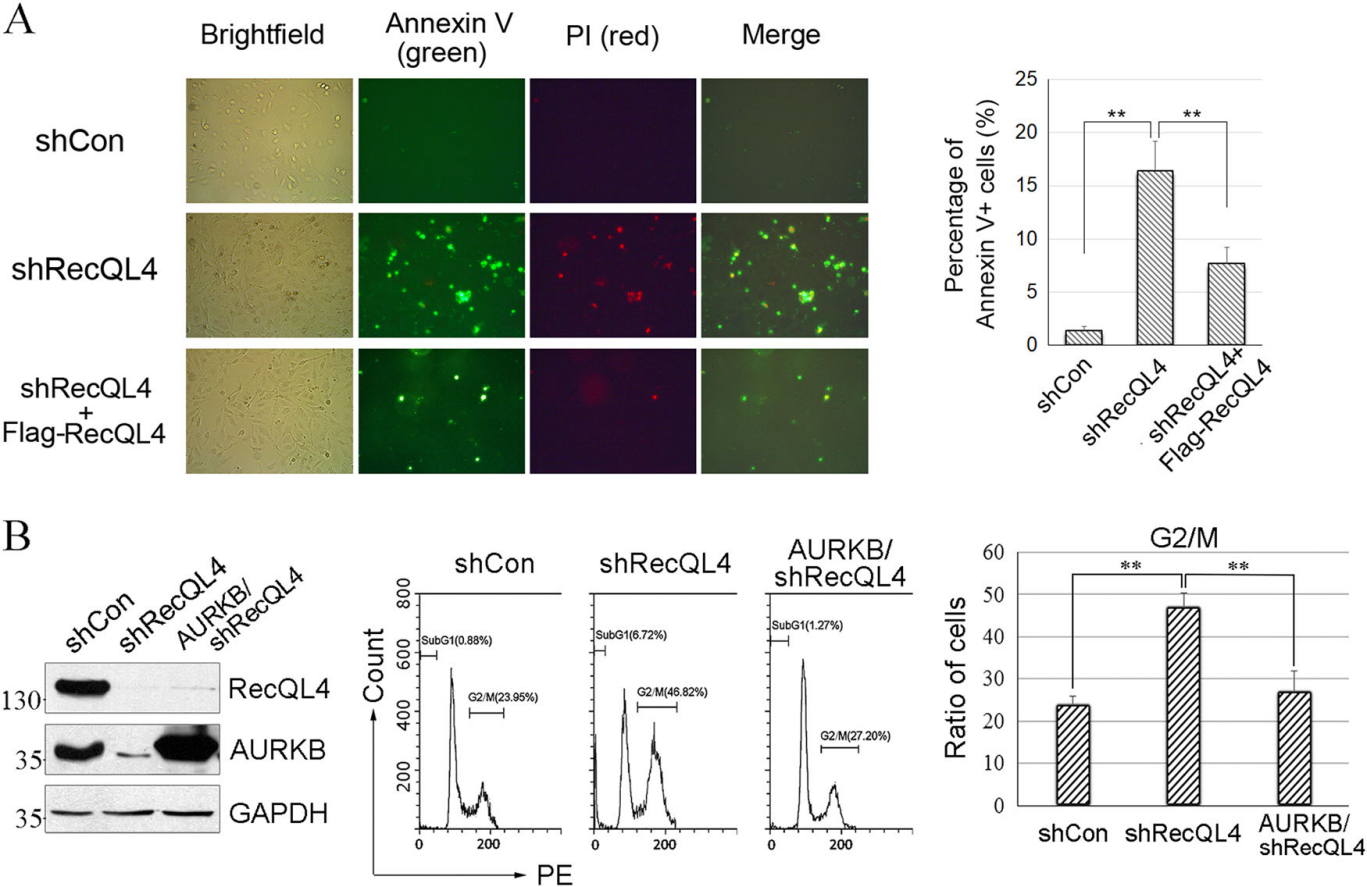
RecQL4 deficiency induced G2/M cell cycle arrest and apoptosis. **a** Apoptotic induction by RecQL4 depletion that was reversed by wild-type RecQL4 reconstitution analyzed by Annexin V + PI staining. To quantify the percentage of Annexin V + PI-positive cells, a total of over 300 cells in six randomly selected fields were counted under a epifluorescence microscope, and the number of positive cells (Annexin V + PI staining) was recorded. Data obtained from three independent experiments are presented as mean percentage ± SD. One-way ANOVA statistical analysis, **p value <0.01. **b** Percentage of the G2/M-phase cells in RecQL4-deficient U2OS cells with or without AURKB reconstitution were measured by PI staining and by flow cytometer at 72 h post transfection. Forced expression of AURKB in RecQL4-suppressed U2OS cells showed the attenuation of cells at the G2/M phase. Data represent the mean ± SD from three independent experiments. One-way ANOVA statistical analysis, **p value <0.01

Given the regulation of AURKB by RecQL4, we wished to determine the impact of RecQL4 expression on cell cycle regulation in U2OS cells transduced with a control vector or shRNA RecQL4 vector. In RecQL4-suppressed U2OS cells, the proportion of cells at the G2/M phase gradually increased from 28.31% to 46.17% after 72 h of culture and then decreased to 29.81% after 96 h. In contrast, U2OS cells transduced with control shRNA vector did not show any noticeable accumulation of cells at the G2/M phase. In addition, RecQL4-suppressed U2OS cells assayed at 96 h showed an elevated sub-G1 population (30.45%), which was significantly higher than cells transduced with shRNA control vector (Fig. S7). In support, using Annexin V plus PI staining assay, the percentage of apoptotic cells was observed to be significantly increased after RecQL4 silencing, and this apoptotic effect could be reversed by reconstitution of wild-type Flag-tagged RecQL4 (Fig. 5a).These findings in agreement with our previous study^18^ suggest that the loss of RecQL4 results in a transient G2/M accumulation followed by a premature exit resulting in cell death.

We next analyzed whether the regulatory role of RecQL4 in the mitotic process is mediated through AURKB. For this purpose, AURKB was ectopically expressed in RecQL4-suppressed U2OS cells and AURKB protein was examined by Western blotting. As shown in Fig. 5b, the proportion of the G2/M-phase cells at 72 h was 23.95% and 46.82% in shRNA Control and shRNA RecQL4-treated cells, respectively. However, ectopic expression of AURKB in RecQL4-suppressed cells reduced the G2/M fraction to 27.20% illustrating that the accumulation of cells occurring in the absence of RecQL4 at the G2/M phase was alleviated by AURKB. Taken together, our results suggest that RecQL4 suppression reduces the viability of cells through downregulation of AURKB by causing the G2/M phase accumulation and premature mitotic exit.

## Discussion

Recent studies have demonstrated that RecQL4 helicase plays crucial roles in DNA replication and diverse DNA repair pathways. Mutations in RecQL4 results in three autosomal recessive disorders: RTS, RAPADILINO syndrome, and BGS^4^. Manifestation of three clinically related human syndromes suggests that RecQL4 is indispensable for genomic instability. Cells of RecQL4-deficient RTS patients display both structural and numerical chromosome alterations involving aneuploidy, mosaicism, and isochromosomes implicating a role for RecQL4 in the maintenance of chromosomal stability^6,7^. In support, RecQL4 knockout mouse cells also displayed aneuploidy with an elevated frequency of chromosomes with premature centromere separation^2^. However, the molecular cause(s) for the chromosomal instability observed in RecQL4-deficient RTS patients remains enigmatic. In this study, we have provided evidence for the first time that RecQL4 physically and functionally interacts with an important mitotic factor, AURKB, and regulates its expression, and RecQL4 helicase domain is critical in modulating AURKB ubiquitination and stability. Furthermore, suppression of RecQL4 reduces the expression of AURKB and results in apoptotic cell death owing to an increased accumulation of mitotic irregularities.

Several lines of evidence suggest that RecQL4 plays an important role in DNA replication initiation and that all of the essential replisome factors (CDC45, MCM2–7 helicases, MCM10, and GINS) interact with RecQL4, and the interaction of CMG complex with RecQL4 is regulated by different phases of cell cycle^4,28^. Further, the N-terminus of RecQL4 was shown to recruit DNA polymerase α to DNA replication initiation sites^29^. RecQL4 through its interaction with Cut5, a homolog of Dpb11, accomplishes the loading of DNA polymerases onto chromatin^29^. Given the vital role for RecQL4 in DNA replication, it is not surprising that the cells of RTS patients with mutations in RecQL4 show a retarded proliferation in vitro. In this study, we provide further line of evidence that RecQL4–AURKB axis is downstream of hTERT and plays an essential role in the acquisition of infinite proliferative potential of human RTS fibroblasts induced by hTERT. RecQL4 has been demonstrated to unwind the telomeric D-loops containing the oxidative lesions possibly through interactions with some of the telomere regulatory proteins, TFR1, TRF2, and POT1^19^. The activation of hTERT has been shown to be an intrinsic event for carcinogenesis^30^. A recent study revealed that AURKB is a novel regulator of TRF1 binding and telomeric integrity^31^. It is likely that hTERT activation leads to RecQL4 upregulation, which in turn can protect the telomere stability in cancer cells both by its unwinding and DNA repair activities, and meanwhile, both RecQL4 and AURKB work in a concerted manner to achieve the telomeric stability in cancer cells.

Elevated RecQL4 expression observed in different human cancers has been demonstrated to correlate with tumorigenic potential in both breast and prostate cancer cells, and cisplatin resistance in gastric cancer cells^26,32^. It is tempting to speculate that the elevated expression of RecQL4 confers survival advantage to cancer cells by protecting them from mitotic irregularities through upregulation of AURKB. In support, we have demonstrated that suppression of RecQL4 in both metastatic prostate^32^ and breast^18^ cancer cells led to retarded proliferation and increased cell death. In corroboration with aberrant elevation of RecQL4 in human cancers, abnormal activation of AURKB was also observed to be a frequent event in human cancer cells^18,33–35^. AURKB belongs to chromosomal passenger complex and is essential for an accurate and equal segregation of chromosomal material during mitosis^36^. Although AURKB has not been established as an oncogene by standard criteria, forced exogenous overexpression of AURKB in Chinese hamster embryo cells results in lagging chromosomes during mitosis and increased invasiveness in vivo, suggesting a role for AURKB in tumorigenesis^37^. Consistently, AURKB was found to be aberrantly activated in cancer types, including multiple myeloma, colorectal, prostate, and pancreatic cancers^33^, and associated with a poor prognosis of cancer patients^34^. In this study, we demonstrated for the first time that AURKB was a downstream target for RecQL4, and RecQL4 elevation stabilized the AURKB protein through inhibiting its ubiquitination-mediated degradation. Consistent with this observation, we showed that RecQL4 suppression led to reduced cell viability and mitotic stability through downregulation of AURKB expression in U2OS cells. In a recent study, USP14, one of the proteasome-associated deubiquitinating enzymes, was found responsible for AURKB stabilization and AURKB stabilization by ectopic expression of USP14 prevented the apoptosis induced by chemotherapeutic drugs in leukemia cells^38^. We recently reported that the resistance to cisplatin treatment stems from the overexpression of RecQL4 in human gastric cancer cells and identified the RecQL4-MDR1-YB axis as a predicting factor for the cellular response to cisplatin. It is not currently known whether or not AURKB stabilization by RecQL4 involves USP14. RecQL4 was shown earlier to interact with ubiquitin ligases UBR1 and UBR2 of the N-end rule pathway^39^, which functions in the regulation of peptide import, chromosomal stability, meiosis, apoptosis, and cardiovascular development. Although RecQL4 was found in the same complex with UBR1 and UBR2, RecQL4 was not ubiquitinylated in vivo^39^. One striking feature that deserves a careful attention is that RecQL4 distribution is predominantly nuclear in non-transformed cells and is cytoplasmic in immortalized cells^39^. It is not clear at this point whether RecQL4 undergoes specific post-translational modifications during the immortalization process, which could explain the differences observed in the localization of RecQL4 in nuclear and cytoplasmic compartments. These interesting aspects have to be investigated further to verify the multifaceted roles of RecQL4 in cancer cells.

Chromosomal abnormalities including trisomy, aneuploidy, and chromosomal rearrangements have been observed in both RTS patient cells and RecQL4-deficient mouse cells^2,6^, but the causative role of RecQL4 for the chromosomal instability was not clearly elucidated. Here, we provide several lines of evidence to demonstrate that RecQL4-mediated regulation of AURKB is essential for a wide range of cellular activities: (I) Cellular proliferation, (II) cell cycle regulation, (III) immortalization, and (IV) mitotic stability. By demonstrating the physical and functional interaction RecQL4 with AURKB, our study throws some light on one of the probable causes for the chromosomal instability observed in RecQL4-deficient RTS patients.

## Materials and methods

### Cell lines and culture

Primary HMEC and human prostate epithelial cells (PHECs) were ordered from from Clonetics BioWhittaker (Walkersville, MD, USA). MCF-10F, RWPE1, U2OS, and 293T cells were ordered from ATCC. MGC-803 cells were obtained from Cell Resource Center, Chinese Academy of Medical Sciences, China. The HMEC/HMEC-hTERT and PHEC/PHEC-hTERT cells were maintained in serum-free mammary (Invitrogen) and prostate (PrEBM, Clonetics BioWhittaker) basal medium supplemented with growth factors, respectively. RWPE1 cells were cultured in keratinocyte serum-free medium (Invitrogen). U2OS and 293T cells were cultured in Dulbecco's modified Eagle's medium (DMEM) containing 10% fetal bovine serum (FBS). MCF-10F cells were cultured in DMEM/F12 (1:1) media containing 5% horse serum (Invitrogen), 10 μg/mL insulin, 20 ng/mL epidermal growth factor, and 500 ng/mL hydrocortisone. MGC-803 cells were cultured in RPMI-1640 supplemented with 10% FBS. All cultures were kept at 37 °C with 5% CO_2_ atmosphere. Mycoplasma was tested as negative using Mycoplasma Detection Kit (Biotool, USA).

### G1-phase synchronization

U2OS cells were synchronized in the G1 phase of cell cycle by DTB. The cells were treated with 2.5 mM thymidine for 14 h and cultured in thymidine-free culture medium for 12 h. The cells were treated second time with thymidine for 12 h and then released into a drug-free medium allowing the cells enter into the S or G2 phase as described^40^.

### Antibodies

The following antibodies were used for this study: rabbit polyclonal anti-RecQL4 (CST #2814, Cell Signaling; NB 25470002, Novus Biologicals); rabbit polyclonal anti-AURKB (CST #3094, Cell Signaling; NB100-294, Novus Biologicals); rabbit anti-phospho-Aurora A (Thr288)/Aurora B (Thr232)/Aurora C (Thr198) (D13A11) XP (#2914, Cell Signaling); mouse anti-caspase-8 monoclonal antibody (CST #9746, Cell Signaling); rabbit anti-Survivin polyclonal antibody (sc-10811, Santa Cruz); mouse anti-Flag monoclonal antibody (F3165, Sigma-Aldrich); mouse anti-GAPDH monoclonal antibody (MB374, Millipore); mouse anti-β-actin monoclonal antibody (sc-47778, Santa Cruz).

### Cloning and protein expression

A DNA insert corresponding to human RecQL4 was amplified and inserted into the *Nde*I and *Xho*I sites of vector pET-16b (Novagen)^26^. The pEBG-gst-AURKB-NT(−), pEBG-gst-AURKB-M(−), and pEBG-gst-AURKB -CT(−) were constructed with vector pEBG^41^. A DNA fragment corresponding to human AURKB amplified by PCR using the primers 5′-CGCGGATCCCTACCATGTCCCCTATACTAGGT-3′ and 5′-CGCGGATCCCAGGGGCCCCTGGAA-3′ was cloned into vector pZeoSV2(+) (Life Technologies). To generate Flag-tagged AURKB full-length, the corresponding DNA fragment was PCR amplified and cloned into pFlag-CMV-4 (Sigma-Aldrich). To generate Flag-tagged full-length and truncated RecQL4, the corresponding DNA fragments were PCR amplified and cloned into vector pcDNA3 (Invitrogen). For RecQL4 shRNA lentiviral constructs, two 21-mer shRNA sequences including shRNA1: GCTCAAGGCCAATCTGAAAGG and shRNA2: GGGAATCTGTCCTGCAGAAGA (366–386 and 1727–1747 bp, accession no. NM_004260.3) and a control scrambled 21-mer shRNA (shControl): GAAGAGGACACGCCTTAGACT were cloned into lentiviral vector and generated lentiviral particles as previously described^42,43^.

Full-length RecQL4 containing N-terminal His tag and C-terminal Flag tag were purified using the method described previously^26,44^. Briefly, proteins were expressed in Rosetta (DE3) pLysS cells and induced with isopropyl-β-d-thio-galactoside. Cells were lysed in the buffer, followed by sonication. The supernatant was clarified by centrifugation and applied to High-Q column, and the flow through was collected and dialyzed against buffer and incubated with Flag M2 beads (Sigma). The M2-bound proteins were washed with buffer, eluted with buffer containing Flag peptide, and dialyzed against buffer (50 mM Tris-HCl, pH 8.0, 10% glycerol, 500 mM KCl, 1 mM EDTA, and 1 mM dithiothreitol).

### GST Pull-down and immunoprecipitation assay

Monoclonal anti-Flag M2 affinity gel (Sigma-Aldrich) and immobilized glutathione (#15160, Thermo Scientific) were used for immunoprecipitation. For each sample, 20–40 μL of 50% gel suspension and 1.0 mg of protein in 1000 μL of lysis buffer (50 mM Tris-HCl, pH 7.5, 150 mM NaCl, 1% NP-40, 5 mM EDTA, 5 mM EGTA, 20 mM NaF, 0.1 mM phenylmethylsulfonyl fluoride, 0.5 mM benzamidine, 1 mg/mL leupeptin, 1 mg/mL aprotinin, 2 mM microcystin, and 0.1 mM NaVO_3_) were used. After rotation at 4 °C for 3–4 h, the agarose gel was washed three times with 1 mL of lysis buffer. The tagged proteins were eluted and heated in 2× sodium dodecyl sulfate (SDS) sample buffer^18^. The immunoprecipitated proteins were detected by Western blotting using antibodies specific for RecQL4, Flag, or AURKB or by SDS-polyacrylamide gel electrophoresis and Coommassie Brilliant Blue staining.

Pull-down of the RecQL4 and purified protein using immobilized glutathione was performed using the following protocol. The bait protein was expressed as a (GST, GST-AURKB) fusion protein in *E. coli* and immobilized on a glutathione resin. Cell lysate with 1 mg of protein in 1000 μL of lysis buffer was prepared by sonicated, with or without DNase I (Sigma-Aldrich, catalog number: DN25) digestion at room temperature for 30 min. Next, the immobilized GST-AURKB was added to the prepared cell lysate and purified RecQL4 protein, and the mixture was rotated at 4 °C for 2 h. The resins were washed, and the bound proteins were eluted with 2× SDS sample buffer, and detected by Western blotting.

### In vivo ubiquitination assays and proteasome inhibition

Ubiquitinated intermediates in human cells were detected with the (His)_6_-tagged Ubi method of Salghetti as reported by Méndez et al.^45^. A total of 1 × 10^6^ U2OS cells with RecQL4 knockdown were transfected with pcDNA3.1-Flag-AURKB in the absence or the presence of plasmids expressing His-tagged ubiquitin and Flag-tagged RecQL4 (pcDNA3.1-Flag-RecQL4). Cells were harvested 36 h post transfection, and His-tagged (therefore ubiquitinated) proteins were purified on Ni-NTA-resin and subjected to Western blotting. To inhibit the proteasome, 1 × 10^6^ U2OS cells with shRecQL4 knockdown were treated for 3 h with MG-132 at a concentration of 10 μM. Flag-tagged proteins were detected by Western blotting with anti-Flag monoclonal antibody.

### Flow cytometry

Both floating and attached cells were collected, washed twice with cold phosphate-buffered saline (PBS), and then fixed by slowly adding 5 mL 75% ice-cold ethanol to the cell suspension dropwisely. After 2 h, the samples were washed twice with PBS and suspended in 300 µL propidium iodide (PI)/Triton X-100 staining PBS buffer with 0.1% (v/v) Triton X-100 (Sigma), 100 µg DNase-free RNase A (Sigma), and 20 µg/mL PI (Sigma) for 30 min at 20 °C. *7*-Aminoactinomycin D (7-AAD) (559925, BD Biosciences) and BeyoClick™ EdU-488 Staining Kit (Cat. No. C0071S) were used to detect cell proliferation. Low toxicity EdU (5-ethynyl-2′-deoxyuridine) incorporation into DNA is an effective replacement for bromodeoxyuridine. The infected U2OS cells were incubated with 10 mM EdU for 30 min just before collecting cells at 48 and 72 h and trypsinized, treated with 4% paraformaldehyde in PBS, washed once with 3% BSA/PBS, resuspended in permeabilization buffer, washed once with BSA buffer, and then incubated with the assay mixture for 30 min, washed once, and then 7-AAD was added to the suspended cells for 10 min. The staining samples were then analyzed in a flow cytometer (CytoFLEX, Beckman Coulter or BD FACSAria II), and the cell cycle distributions were then quantitatively analyzed. All data analysis was done with the CytExpert software (Beckman Coulter).

### Immunofluorescence staining for mitotic phase cells

U2OS cells were seeded onto chamber slides for 24 h and then infected with shControl and shRecQL4 adenovirus for 48 h. After washing with PBS and fixed in 4% paraformaldehyde buffer, the cells were permeabilized with 0.1% Triton X-100 for 10 min and then blocked with 2% BSA (Santa Cruz) in PBS. For analysis of the mitotic phase cells, the cells were incubated with rabbit anti-AURKB antibody (1:150 dilution, Novusbio) at 4 °C overnight. After washing with PBS for three times, cells were incubated with fluorescein goat anti-rabbit IgG antibody (1:200 dilution, Vector Laboratories, FI-1000) at room temperature for 1 h, and then washed with PBS for three times. Then, the cells were incubated with mouse anti-β-tubulin antibody (1:200 dilution, Sigma, T5293) at 4 °C overnight. After washing with PBS three times, cells were incubated with DyLight 594 horse anti-mouse IgG antibody (Vector Laboratories, DI-2594) at room temperature for 1 h, and counterstained with DAPI. The mitotic phase cells were visualized under a Leica confocal laser scanning microscope TCS SP.

For p-H3 staining, the cells were immunostained with rabbit p-Histone H3 (Ser10, D2C8 clone) monoclonal antibody (1:200 dilution, Cell signaling, #3377) at 4 °C overnight, and then incubated with Texas Red^®^ goat anti-rabbit IgG antibody (Vector, TI-1000) and counterstained with DAPI. The p-H3-positive cells were quantified by Flow Cytometry.

### Analysis of apoptotic cells

Quantitation of apoptotic cells under control and RecQL4 knockdown conditions was done using the Annexin V-FITC/PI Detection Kit (Beyotime, Cat. No. C1063) according to the manufacturer’s protocol. To quantify the percentage of Annexin V + PI-positive cells, a total of over 300 cells in six randomly selected fields were counted under a fluorescence microscope, and the number of cells with Annexin V + PI-positive staining was recorded. Data were represented as mean percentage ± SD from three independent experiments. One-way analysis of variance (ANOVA) statistical analysis. *P* value <0.05 was considered as statistically significant.

## Electronic supplementary material

Figure S1

Figure S2

Figure S3

Figure S4

Figure S5

Figure S6

Figure S7
