# Supplementary figures and images for: RecQL4-Aurora B kinase axis is essential for cellular proliferation, cell cycle progression, and mitotic integrity
Source: Oncogenesis. 2018 Sep 12;7(9):68. doi: 10.1038/s41389-018-0080-4 (PMC6134139; doi:10.1038/s41389-018-0080-4)

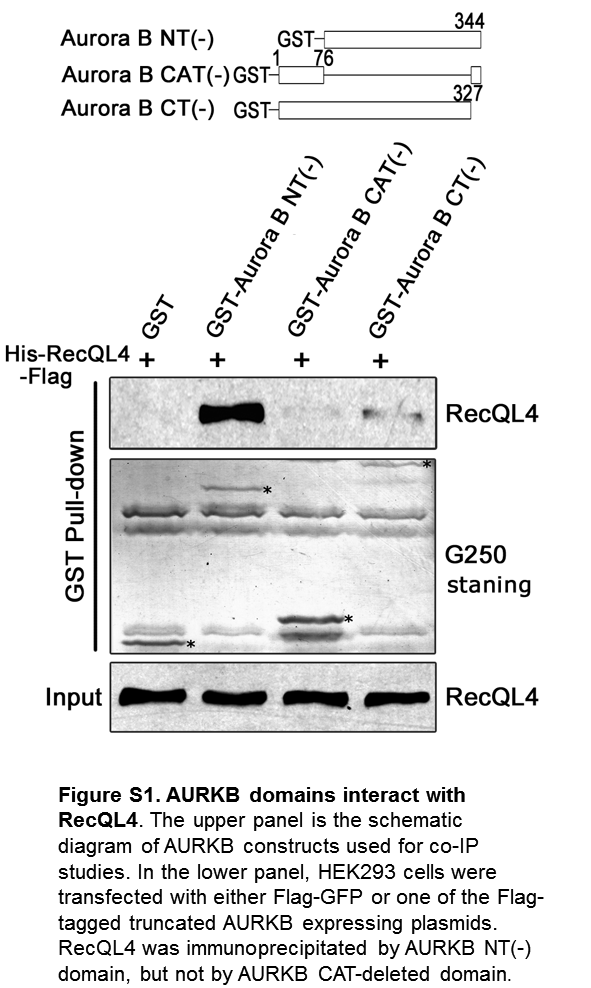

Supplement: Supplementary file 1 — Figure S1 [file 41389_2018_80_MOESM1_ESM.tif]

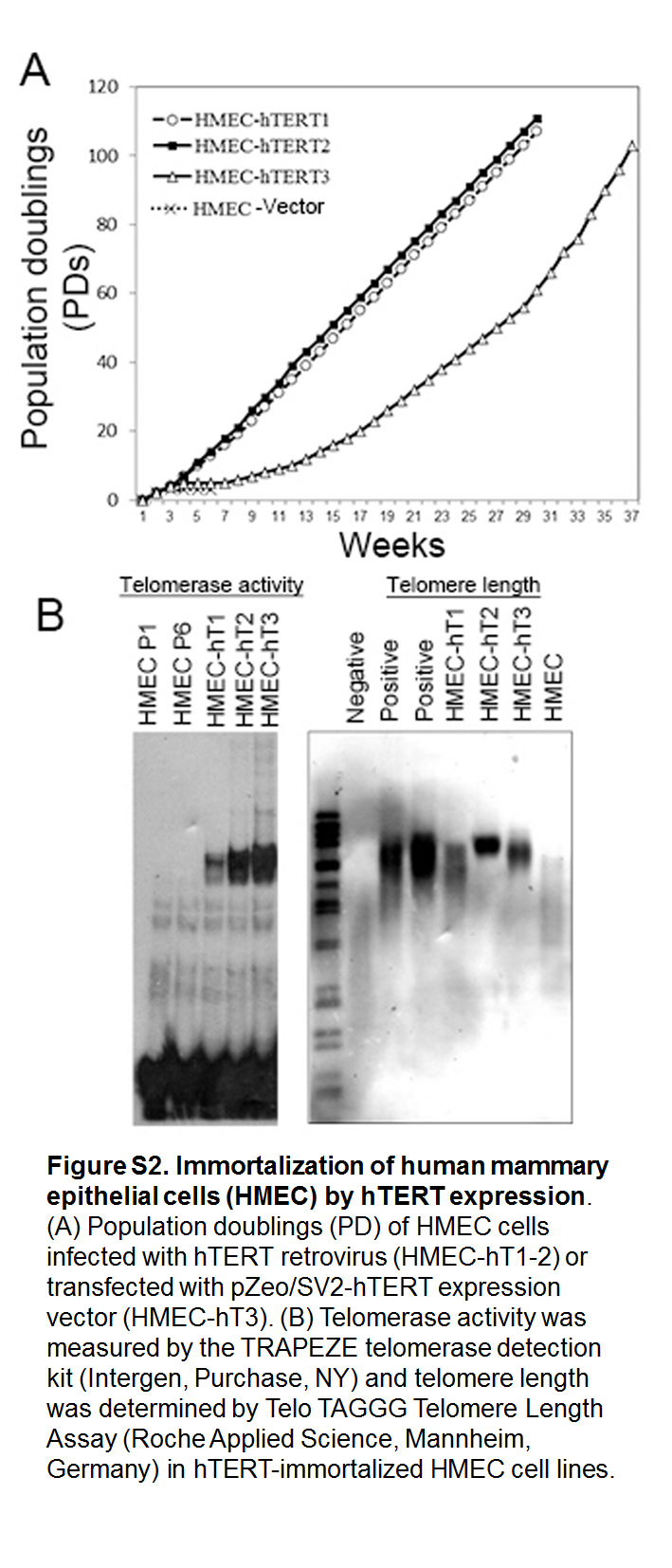

Supplement: Supplementary file 2 — Figure S2 [file 41389_2018_80_MOESM2_ESM.tif]

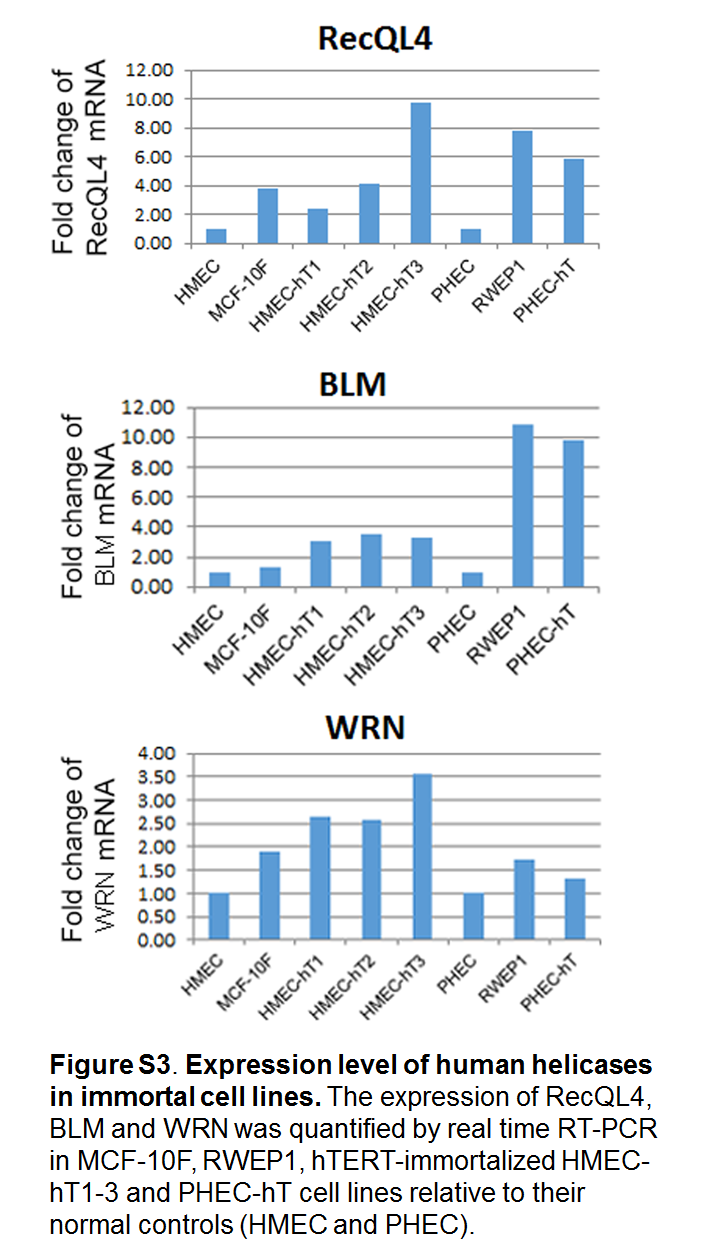

Supplement: Supplementary file 3 — Figure S3 [file 41389_2018_80_MOESM3_ESM.tif]

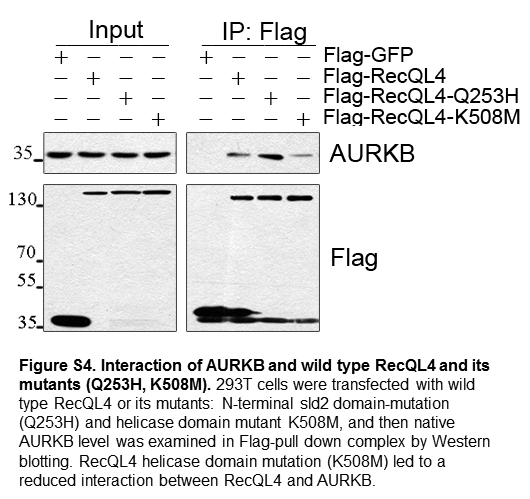

Supplement: Supplementary file 4 — Figure S4 [file 41389_2018_80_MOESM4_ESM.tif]

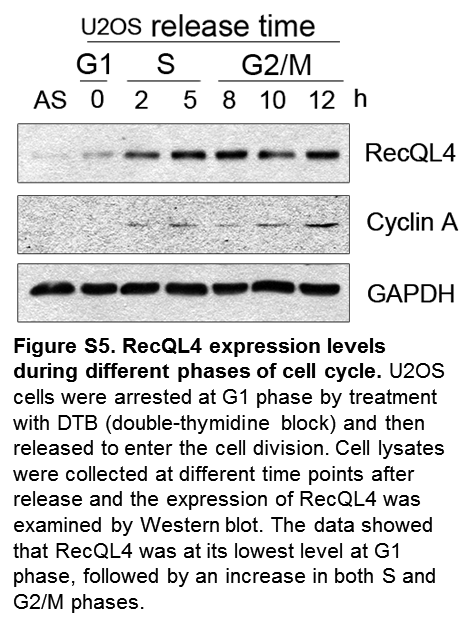

Supplement: Supplementary file 5 — Figure S5 [file 41389_2018_80_MOESM5_ESM.tif]

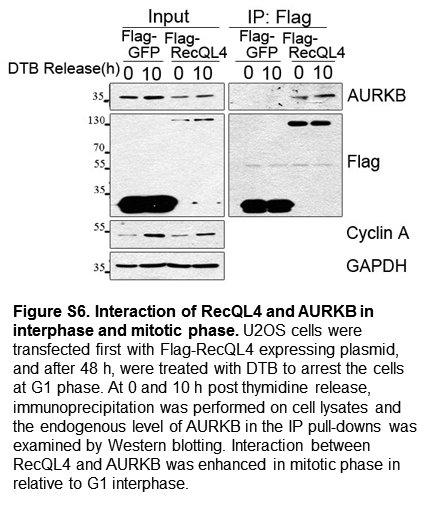

Supplement: Supplementary file 6 — Figure S6 [file 41389_2018_80_MOESM6_ESM.tif]

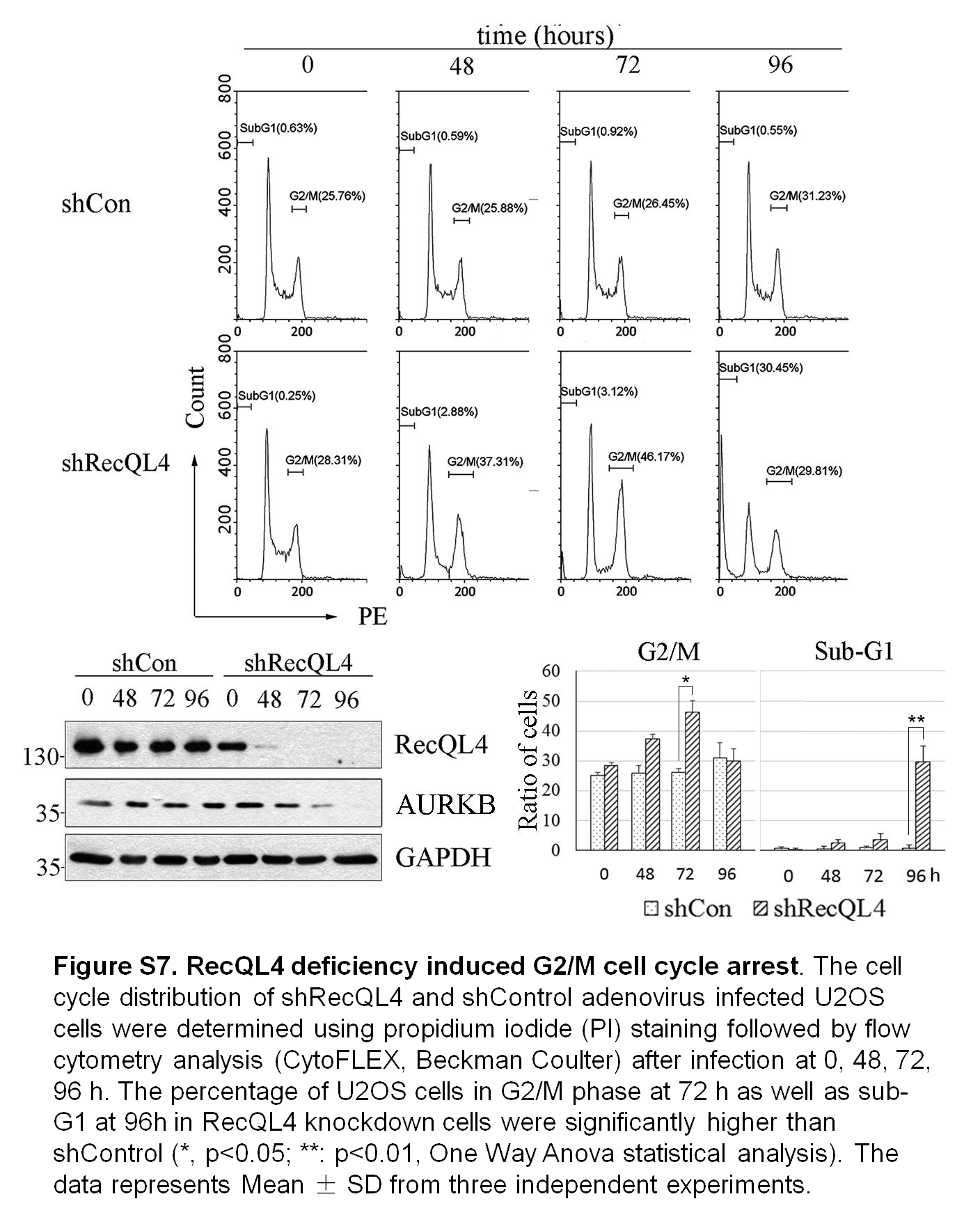

Supplement: Supplementary file 7 — Figure S7 [file 41389_2018_80_MOESM7_ESM.tif]
